# Supplementary material for: Novel Survival Features Generated by Clinical Text Information and Radiomics Features May Improve the Prediction of Ischemic Stroke Outcome
Source: Diagnostics (Basel). 2022 Jul 8;12(7):1664. doi: 10.3390/diagnostics12071664 (PMC9324145; doi:10.3390/diagnostics12071664)
Supplement: Supplementary file 1 [file diagnostics-12-01664-s001.zip › S1ú▌The selected radiomics features from the 13 methods and the features in three classification situations.pdf]

Definition	Group	Radiomics Features	Method	mRS_2	mRS_4	mRS_7
F1	First-order	original_firstorder_10Percentile_0	CMIM; DISR; JMI; MIFS; MIM; MRMR; Alpha;	1	0	1
F2	Wavelet	wavelet-HHL_glszm_GrayLevelNonUniformityNormalized_0	CMIM; JMI; MIFS;	0	0	0
F3	Wavelet	wavelet-HHL_glszm_LowGrayLevelZoneEmphasis_0	CMIM;	0	0	0
F4	Wavelet	wavelet-HHL_glszm_GrayLevelVariance_1	CMIM;	0	0	0
F5	Wavelet	wavelet-HHL_glszm_HighGrayLevelZoneEmphasis_11	CMIM;	0	0	0
F6	Wavelet	wavelet-HHL_glszm_GrayLevelNonUniformityNormalized_12	CMIM;	0	0	0
F7	Wavelet	wavelet-HHL_glszm_GrayLevelVariance_17	CMIM;	0	0	0
F8	First-order	original_firstorder_90Percentile_0	CMIM; DISR; MIM;	0	0	0
F9	First-order	original_firstorder_Kurtosis_0	CMIM; DISR; MIM; Alpha;	0	0	0
F10	First-order	original_firstorder_MeanAbsoluteDeviation_0	CMIM; DISR; MIM;	0	0	0
F11	First-order	original_firstorder_Mean_0	CMIM; DISR; MIM;	0	0	0
F12	First-order	original_firstorder_Median_0	CMIM; DISR; MIM;	0	0	0
F13	First-order	original_firstorder_RootMeanSquared_0	CMIM; DISR; MIM;	0	0	0
F14	First-order	original_firstorder_Skewness_0	CMIM; DISR; MIM;	0	0	0
F15	First-order	original_firstorder_Variance_0	CMIM; DISR; MIM;	0	0	0
F16	GLCM	original_glcm_Autocorrelation_0	CMIM; DISR; MIM;	0	0	0
F17	GLCM	original_glcm_ClusterProminence_0	CMIM; DISR; MIM; Alpha;	0	0	0
F18	GLCM	original_glcm_ClusterShade_0	CMIM; DISR; MIM;	0	0	0
F19	GLCM	original_glcm_ClusterTendency_0	CMIM; DISR; MIM;	0	0	0
F20	GLCM	original_glcm_Correlation_0	CMIM; DISR; MIM;	0	1	0
F21	GLCM	original_firstorder_Minimum_0	DISR; MIM;	0	0	0
F22	GLCM	original_firstorder_Range_0	DISR; MIM;	0	0	0
F23	GLCM	original_glcm_DifferenceEntropy_0	DISR; MIM;	0	0	1
F24	GLCM	original_glcm_DifferenceVariance_0	DISR; MIM;	0	0	0
F25	GLCM	original_glcm_JointAverage_0	DISR; MIM;	0	0	0
F26	GLCM	original_glcm_MCC_0	DISR; MIM;	0	0	0
F27	Wavelet	wavelet-HHH_glszm_GrayLevelNonUniformityNormalized_0	JMI; MIFS; MRMR;	0	0	0
F28	Wavelet	wavelet-HHH_glszm_GrayLevelNonUniformityNormalized_20	JMI;	0	0	0
F29	Wavelet	wavelet-HHH_glszm_GrayLevelNonUniformityNormalized_11	JMI;	0	0	0
F30	Wavelet	wavelet-HHL_glszm_GrayLevelNonUniformityNormalized_18	JMI;	0	0	0
F31	Wavelet	wavelet-HHL_glszm_HighGrayLevelZoneEmphasis_1	JMI;	0	0	0
F32	Wavelet	wavelet-HHL_glszm_HighGrayLevelZoneEmphasis_13	JMI;	1	1	1
F33	Wavelet	wavelet-HHL_glszm_HighGrayLevelZoneEmphasis_3	JMI; MRMR;	0	0	0
F34	Wavelet	wavelet-HHH_glszm_GrayLevelNonUniformityNormalized_16	JMI;	0	0	0
F35	Wavelet	wavelet-HHH_glszm_HighGrayLevelZoneEmphasis_18	JMI;	0	1	0
F36	Wavelet	wavelet-HHL_glszm_GrayLevelNonUniformityNormalized_19	JMI;	0	0	0
F37	Wavelet	wavelet-HHL_glszm_GrayLevelNonUniformityNormalized_47	JMI;	0	0	0
F38	Wavelet	wavelet-HHH_glszm_SizeZoneNonUniformity_0	JMI;	0	0	0
F39	Wavelet	wavelet-HHL_glszm_LowGrayLevelZoneEmphasis_47	JMI;	0	0	0
F40	Wavelet	wavelet-HHL_glszm_LowGrayLevelZoneEmphasis_41	JMI;	0	0	0
F41	Wavelet	wavelet-HHL_glszm_SizeZoneNonUniformity_2	JMI;	0	0	0
F42	Wavelet	wavelet-HHL_glszm_HighGrayLevelZoneEmphasis_37	JMI;	0	0	0
F43	Wavelet	wavelet-HHH_glszm_GrayLevelVariance_3	JMI; MIFS;	1	1	1
F44	Wavelet	wavelet-HHL_glszm_HighGrayLevelZoneEmphasis_34	JMI;	0	0	0
F45	Wavelet	wavelet-HHL_glszm_HighGrayLevelZoneEmphasis_9	MIFS; MRMR;	0	0	0
F46	Wavelet	wavelet-HHH_glszm_GrayLevelNonUniformityNormalized_3	MIFS; MRMR;	0	0	0
F47	Wavelet	wavelet-HHH_glszm_GrayLevelVariance_2	MIFS; MRMR;	1	1	1
F48	Wavelet	wavelet-HHH_glszm_GrayLevelNonUniformityNormalized_2	MIFS; MRMR;	0	0	0
F49	Wavelet	wavelet-HHL_glszm_GrayLevelVariance_46	MIFS; MRMR;	0	0	0
F50	Wavelet	wavelet-HHH_glszm_GrayLevelVariance_0	MIFS; MRMR;	0	0	0
F51	Wavelet	wavelet-HHL_glszm_GrayLevelVariance_3	MIFS;	0	0	0
F52	Wavelet	wavelet-HHL_glszm_GrayLevelNonUniformityNormalized_6	MIFS; MRMR;	0	0	0
F53	Wavelet	wavelet-HHH_glszm_HighGrayLevelZoneEmphasis_3	MIFS; MRMR;	1	1	0
F54	Wavelet	wavelet-HHL_glszm_GrayLevelVariance_6	MIFS;	0	0	0
F55	Wavelet	wavelet-HHL_glszm_GrayLevelVariance_15	MIFS; MRMR;	0	0	0
F56	Wavelet	wavelet-HHL_glszm_GrayLevelNonUniformityNormalized_3	MIFS;	0	0	0
F57	Wavelet	wavelet-HHL_glszm_GrayLevelNonUniformityNormalized_49	MIFS;	0	0	0
F58	Wavelet	wavelet-HHL_glszm_GrayLevelNonUniformityNormalized_36	MIFS;	1	0	1
F59	Wavelet	wavelet-HHH_glszm_HighGrayLevelZoneEmphasis_10	MRMR;	0	0	0
F60	Wavelet	wavelet-HHL_glszm_HighGrayLevelZoneEmphasis_14	MRMR;	0	0	0
F61	Wavelet	wavelet-HHL_glszm_HighGrayLevelZoneEmphasis_0	MRMR;	0	0	0
F62	Wavelet	wavelet-HHL_glszm_LowGrayLevelZoneEmphasis_9	MRMR;	0	0	0
F63	Wavelet	wavelet-HHH_glszm_GrayLevelNonUniformityNormalized_12	MRMR;	0	0	0
F64	Wavelet	wavelet-HHL_glszm_GrayLevelVariance_44	MRMR;	0	0	0
F65	Log-sigma	log-sigma-1-0-mm-3D_firstorder_Mean_20	Fisher; ReliefF; Alpha; FS; TS;	0	0	0
F66	Log-sigma	log-sigma-2-0-mm-3D_firstorder_Mean_20	Fisher; ReliefF; FS; TS;	0	0	0
F67	Log-sigma	log-sigma-1-0-mm-3D_firstorder_Mean_21	Fisher; ReliefF; FS; TS;	0	0	0
F68	Log-sigma	log-sigma-2-0-mm-3D_firstorder_Mean_21	Fisher; ReliefF; FS; TS;	0	0	0
F69	Wavelet	wavelet-LLH_firstorder_Variance_17	LS;	0	0	0
F70	Log-sigma	log-sigma-2-0-mm-3D_firstorder_90Percentile_20	LS;	0	0	0

F71	Wavelet	wavelet-LHH_gldm_GrayLevelVariance_25	LS;	0	0	0
F72	Wavelet	wavelet-LHH_firstorder_RootMeanSquared_31	LS;	0	0	0
F73	GLCM	original_glcm_SumSquares_39	LS;	0	0	0
F74	First-order	original_firstorder_90Percentile_48	LS;	0	0	0
F75	Log-sigma	log-sigma-1-0-mm-3D_firstorder_Mean_17	ReliefF;	1	0	0
F76	Log-sigma	log-sigma-2-0-mm-3D_firstorder_Mean_17	ReliefF;	0	0	0
F77	Log-sigma	log-sigma-1-0-mm-3D_firstorder_Mean_18	ReliefF; Lasso;	0	0	0
F78	Log-sigma	log-sigma-2-0-mm-3D_firstorder_Mean_18	ReliefF;	0	0	0
F79	Log-sigma	log-sigma-1-0-mm-3D_firstorder_Mean_19	ReliefF; Lasso; TS;	0	0	0
F80	Log-sigma	log-sigma-2-0-mm-3D_firstorder_Mean_19	ReliefF; FS; TS;	0	0	0
F81	Log-sigma	log-sigma-3-0-mm-3D_firstorder_Mean_19	ReliefF;	0	0	0
F82	Wavelet	wavelet-LHL_firstorder_Mean_19	ReliefF;	0	0	0
F83	Log-sigma	log-sigma-1-0-mm-3D_firstorder_Skewness_0	Alpha;	0	1	1
F84	Wavelet	wavelet-LHH_firstorder_Median_0	Alpha;	0	0	0
F85	Log-sigma	log-sigma-1-0-mm-3D_firstorder_Skewness_14	Alpha;	1	0	0
F86	Wavelet	wavelet-LHL_firstorder_Median_14	Alpha;	0	0	0
F87	Log-sigma	log-sigma-1-0-mm-3D_glrlm_LongRunEmphasis_20	Alpha;	0	0	1
F88	Wavelet	wavelet-LHL_firstorder_Mean_3	Alpha;	0	1	1
F89	GLRLM	original_glrlm_LongRunEmphasis_33	Alpha;	0	0	0
F90	Wavelet	wavelet-LLL_firstorder_Kurtosis_0	Lasso;	0	0	0
F91	Wavelet	wavelet-LLL_glrlm_LongRunHighGrayLevelEmphasis_0	Lasso;	1	0	1
F92	Wavelet	wavelet-LLL_gldm_LargeDependenceHighGrayLevelEmphasis_[illegible]	Lasso;	0	0	0
F93	Wavelet	wavelet-HLL_firstorder_Minimum_16	Lasso;	0	1	1
F94	Wavelet	wavelet-HLL_firstorder_Median_17	Lasso;	1	1	0
F95	Wavelet	wavelet-HHL_firstorder_Skewness_17	Lasso;	0	0	0
F96	Wavelet	wavelet-LHL_glcm_Imc2_18	Lasso;	0	0	0
F97	Log-sigma	log-sigma-2-0-mm-3D_firstorder_Skewness_19	Lasso;	0	0	0
F98	Wavelet	wavelet-HLL_firstorder_Median_19	Lasso;	0	0	0
F99	First-order	original_firstorder_Energy_20	Lasso;	1	1	1
F100	Log-sigma	log-sigma-1-0-mm-3D_glcm_MaximumProbability_20	Lasso;	1	0	0
F101	Wavelet	wavelet-HLL_firstorder_Skewness_21	Lasso;	0	0	1
F102	GLCM	original_glcm_MaximumProbability_26	Lasso;	0	0	0
F103	First-order	wavelet-LHH_firstorder_Median_45	Lasso;	0	0	0
F104	Log-sigma	log-sigma-2-0-mm-3D_glszm_ZoneVariance_18	MCFS	0	0	0
F105	Log-sigma	log-sigma-2-0-mm-3D_glszm_LargeAreaEmphasis_18	MCFS	0	0	0
F106	Log-sigma	log-sigma-4-0-mm-3D_firstorder_Mean_35	MCFS	0	0	0
F107	Log-sigma	log-sigma-4-0-mm-3D_firstorder_Mean_36	MCFS	0	0	0
F108	Log-sigma	log-sigma-4-0-mm-3D_firstorder_Mean_34	MCFS	1	1	1
F109	Log-sigma	log-sigma-2-0-mm-3D_gldm_GrayLevelNonUniformity_17	MCFS	0	0	0
F110	GLCM	original_glcm_DifferenceVariance_9	MCFS	0	0	0
F111	GLCM	original_glcm_DifferenceVariance_10	MCFS	0	0	0
F112	Log-sigma	log-sigma-2-0-mm-3D_gldm_GrayLevelNonUniformity_16	MCFS	0	0	0
F113	Log-sigma	log-sigma-2-0-mm-3D_gldm_GrayLevelNonUniformity_18	MCFS	0	0	0
F114	GLCM	original_glcm_DifferenceVariance_8	MCFS	0	0	0
F115	Log-sigma	log-sigma-1-0-mm-3D_gldm_LargeDependenceEmphasis_12	MCFS	0	0	0
F116	Wavelet	wavelet-HLL_firstorder_Entropy_15	MCFS	0	0	0
F117	Log-sigma	log-sigma-4-0-mm-3D_firstorder_Mean_49	MCFS	0	0	0
F118	Log-sigma	log-sigma-1-0-mm-3D_gldm_LargeDependenceEmphasis_13	MCFS	0	0	0
F119	Log-sigma	log-sigma-1-0-mm-3D_glrlm_RunPercentage_12	MCFS	0	0	0
F120	Wavelet	wavelet-HLL_firstorder_Entropy_14	MCFS	0	0	0
F121	GLDM	original_gldm_LargeDependenceHighGrayLevelEmphasis_13	MCFS	0	0	0
F122	Wavelet	wavelet-HLL_firstorder_Entropy_16	MCFS	0	0	0
F123	GLDM	original_gldm_LargeDependenceHighGrayLevelEmphasis_14	MCFS	0	0	0
F124	Log-sigma	log-sigma-1-0-mm-3D_firstorder_Mean_22	FS; TS;	0	0	0
F125	Log-sigma	log-sigma-2-0-mm-3D_firstorder_Mean_22	FS; TS;	1	0	1
F126	Log-sigma	log-sigma-3-0-mm-3D_firstorder_Mean_20	TS;	0	0	0
F127	Wavelet	wavelet-HLL_firstorder_Mean_20	TS;	0	0	0
F128	Wavelet	wavelet-HLL_firstorder_Mean_21	TS;	0	0	0
